# High-dose interleukin2 – a 10-year single-site experience in the treatment of metastatic renal cell carcinoma: careful selection of patients gives an excellent outcome

**DOI:** 10.1186/s40425-016-0174-5

**Published:** 2016-10-18

**Authors:** S. Chow, V. Galvis, M. Pillai, R. Leach, E. Keene, A. Spencer-Shaw, A. Shablak, J. Shanks, T. Liptrot, F. Thistlethwaite, R. E. Hawkins

**Affiliations:** 1 The Christie NHS Foundation Trust, Manchester, UK; 2 The University of Manchester, Manchester, UK; 3 The Christie Clinic, Manchester, UK

**Keywords:** High dose interleukin2, Cytokine therapy, Metastatic renal cell carcinoma, Pathology selection criteria

## Abstract

**Background:**

VEGF-targeted therapy has become the mainstay of treatment for majority of mRCC patients. For most patients, benefit is short-lived and therefore treatment remains palliative in intent. HD IL2 is an effective immunotherapy treatment capable of durable remission in some patients but its unselected use has been difficult due to its modest response rate and considerable adverse effects. Using set pathology criteria as a selection tool in clinical practice, we have been able to show improved outcomes in our previous report. Here, we present an updated and extended report of this treatment and seek to explore any pathological, clinical and treatment variables likely to predict better outcomes.

**Methods:**

This is an extension of a previously reported clinical audit, which includes mRCC cases treated with HD IL2 between 2003 and 2013. Since 2006, tumour specimens of potential candidates were routinely reviewed prospectively and stratified into *Favourable* or *Other* categories based on constitution of histological growth pattern, namely alveolar or solid versus papillary and/or sarcomatoid architecture; clear cell versus granular cell cytoplasmic morphology. HD IL2 was preferentially offered to patients with *Favourable* pathology. Outcome evaluation includes response rates, survival, and treatment tolerance. Multivariate analysis was performed to explore potential prognostic and predictive factors.

**Results:**

Among prospectively selected patients with *Favourable* pathology (*n* = 106), overall response rate was 48.1 % (51/106) with CR rate of 21.6 % (23/106). Median OS was 58.1 months. Factors associated with significantly better response and/or survival includes favourable pathology pattern, higher cycle 1 tolerance and lower number of metastatic organ sites (<3). CAIX (Carbonic anhydrase 9) has prognostic value but is not predictive of response. Toxicities were those expected of IL2 but were manageable on general medical wards, with no treatment-related death. Importantly most complete responses were durable with 76 % (23/30) cases remained relapse-free (median 39 months follow up) and 2 of the seven who relapsed had had long-term disease free survival after resection of oligometastatic relapse.

**Conclusions:**

Our experience shows that HD IL2 remains an effective and safe treatment in well-selected cases of mRCC. The result in this single-institution patient series confirms similar outcomes to our previously reported retrospective series. Given the prospect of long-term remission, fit patients with *Favourable* histology and low disease burden should be considered for HD IL2 in an experienced centre. Better understanding has been gained from this in-depth analysis especially the examination of possible response predictors and strategies that can improve treatment outcome.

**Electronic supplementary material:**

The online version of this article (doi:10.1186/s40425-016-0174-5) contains supplementary material, which is available to authorized users.

## Introduction

Renal cell carcinoma (RCC) is associated with high mortality, with approximately one third of patients diagnosed with advanced or metastatic disease at presentation. Even in patients with localised disease, relapse rate is as high as 40 % despite initial curative surgery and often with disseminated distribution [1, 2]. There have been impressive strides in the management of patients with mRCC using targeted therapies in recent years. However, these treatments remain palliative in intent with little prospect for durable response [3–5]. The recent finding of survival benefit with Nivolumab, a PD1 (Programmed cell death 1) checkpoint inhibitor, compared to Everolimus in second line or more setting [6] represents an exciting development in the treatment paradigm of mRCC. The complete response rate was, however, disappointing (1 %). Longer term survival data including the extension of its use in frontline setting as well as that of other similar agents and combinations will inform us of the true potential of this newly emerged treatment option.

High-dose Interleukin 2 (HD IL-2) is well documented as an agent capable of achieving durable complete response in patients with mRCC [7]. The response rate in unselected populations is only modest with ORR of 14–20 % and CRR of 5–8 % [8–10]. In our centre, we have placed a major emphasis on patient selection using tumour morphology, which has been implemented since its possible therapeutic relevance was reported by Upton and group. This is likely the main factor which has resulted in better outcome as demonstrated in our first report [10]. Herein, we present the updated and extended result of first-line HD IL2 treatment in mRCC patients treated during a 10-year period since 2003. We also aimed to explore any pathological, clinical and treatment variables likely to predict treatment outcome.

## Methods

### Patients

At consideration of HD IL2 treatment, all patients were assessed according to clinical selection criteria (Table 1). Patients with active brain metastasis or those with an autoimmune disorder requiring long-term steroids were excluded. All patients had satisfactory baseline organ function (Creatinine < 1.5 × ULN; ALT/AST < 3 × ULN). A pathological classification was proposed by Upton et al. in 2005 based on histological characteristic which appeared to be predictive of IL2 treatment response [11]. Tumour of clear cell histology with no papillary features, ≤ 50 % granular features, and ≥ 50 % alveolar features were linked to higher responses. Our local retrospective review was similar with regards to favourable alveolar feature but a good proportion of responses were seen among those considered unfavourable by Upton’s criteria namely in those with limited focal papillary component (<10 %), those with >50 % granular cells and those with >50 % solid architecture. These were subsequently adapted in our pathology selection criteria and have been prospectively applied in our clinical practice since 2006. As we are a referral center, all cases were initially diagnosed at peripheral centers. It is standard practice to carry out central pathology review on all external histological tissues by our resident pathologists prior to treatment consideration and stratify into *Favourable* or *Other* categories (Table 2).

**Table 1:** Clinical selection criteria for treatment of HD IL2

Clinical Selection criteria:	
• Histological diagnosis of clear-cell type metastatic renal cell carcinoma with measurable disease • Performance status 0–1 • Prior-nephrectomy • No concomitant use of steroids • No evidence of active CNS involvement • No history of coronary artery disease or normal stress echocardiogram for patient older than 55-year-old of age.

**Table 2:** Prospective pathology-based selection criteria

Type	Histological features
Favourable	Less than 10 % papillary histology, and at least one favourable feature of: • >50 % alveolar and or >50 % solid architecture • <50 % granular cytoplasm or >50 % clear cell features
Other	Histology features other than *Favourable*

Material from a nephrectomy specimen was preferred but if this was not available, analysis of tumour from a metastatic site was used. HD IL2 was offered to patients in the *Favourable* category. Motivated patients with *Other* pathology category type were offered treatment after full informed discussion regarding risk and benefit and other available treatment options at the time.

Note: Individual tumour morphology component fulfilling the percentage threshold of our pathology criteria was termed ‘favourable’, and ‘unfavourable’ if otherwise (non-italic). For example, tumour with >50 % alveolar component is termed ‘favourable alveolar’. Likewise, ‘unfavourable granular and unfavourable papillary’ feature refer to >50 and >10 % of total tumour constituent respectively.

### Membrane carbonic anhydrase 9 (CAIX) expression

Membrane CAIX immunohistochemistry expression has been suggested to be associated with better survival as well as a potential predictor of HD IL-2 response [12–14]. To explore this potential property, CAIX expression was assessed as part of prospective pathology review but was not used as selection criteria. Paraffin-embedded tissue sections were subjected to immunohistochemistry using a mouse monoclonal antibody for CAIX (Novus Biologicals, used at a dilution of 1/2000) and Menarini Intellipath automated immunostainer using Menarini detection system with a DAB chromogen. This was subsequently evaluated and the percentage of tumour cells showing surface membrane positivity on the section was provided.

### High-dose interleukin 2

Each patient received intravenous Interleukin-2 at 600,000 unit/kg given over 15 min at a minimum of 8 hourly intervals up to a maximum of 14 doses as tolerated over a 5-day period. The 5-day treatment session is repeated after a 10-day break. Treatment was delayed or interrupted according to standard guidelines, and full supportive medical measures were implemented as per published guideline [15] and local protocol. Two 5-day treatments constitute one cycle. Depending on therapeutic benefit and tolerance, treatment was repeated every 12 weeks to maximum response. Further cycle(s) are offered in the event of any response after cycle one. Patients with stable disease (SD) may be considered for further treatment especially in disease that was obviously progressing before HD IL2. One further treatment cycle was given after any best overall response (including after CR) if possible. Treatment was discontinued in event of unequivocal progressive disease (PD) or unacceptable toxicity – in addition, treatment was discontinued for stable disease after 2 or more cycles as the patient was unlikely to achieve a complete remission. Written consent for treatment were obtained from patients prior to start of treatment as required by standard practice.

### Outcome measurements and statistical analysis

The primary outcome measurement is overall response rate. Secondary outcomes are survival, treatment tolerance and toxicity. Treatment response was evaluated by CT (computed topography) scan every 12 weeks using RECIST criteria. Overall survival (OS) was calculated from start of treatment to death or censored at time last known to be alive respectively. Progression free survival (PFS) was not an outcome measure in this report due to 2 limitations. Firstly, patients who achieved less than complete responses may have very variable course of subsequent management. PFS in relation to HD IL2 in such instances will be as a result of the effects of both HD IL2 and their subsequent therapy. Secondly, a significant number of patients were referred from distant centres in the country and information regarding PFS, was not easily or accurately obtainable within the resources of this clinical audit once patient’s care has been transferred back to local centre for further management. Last date of observation was 30th October 2015. Response rate modelling was done using logistic regression. Survival analysis was done using a Cox proportional hazard model. Non-linearity of continuous variables (CAIX and Cycle 1 dose) was investigated using a penalized spline basis. All statistical analysis was done using R version 3.2.0 (R Core Team, 2015) and the survival package (v2.38-1, Therneau, 2015). Modeling was, to some extent, exploratory and as there was no control group any effects found here have the potential to be affected by unmeasured confounders. As the treatment population was considered fairly homogenous, the bias on the dose response of this was assumed to be small.

## Results

### Patient population

**Fig. 1:**
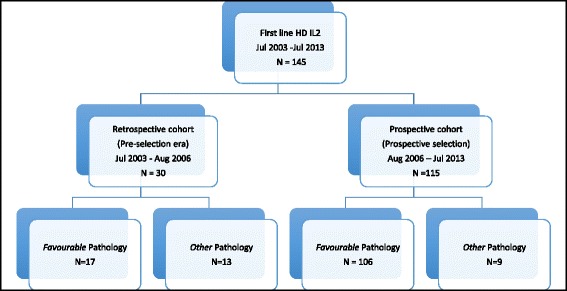
Flow diagram showing cohorts of patient population

This report included a total of 145 mRCC patients who received HD IL2 in the first-line setting from 2003 to 2013 (Fig. 1). Seventy-three additional patients were included since the conclusion of previous review in 2008. Patients with *Favourable* pathology constitute approximately 12 % of all mRCC cases in our centre during this period. Patient characteristic and demographic is as outlined in Table 3. One patient had non-clear cell tumour variant (Papillary type II) whereas the rest had clear cell renal cell carcinoma. Median follow-up duration was 39 months. All patients had ECOG (Eastern Cooperative Oncology Group) performance status 0–1 and had good or intermediate MSKCC (Memorial Sloan-Kettering Cancer Centre) risk score. The commonest site of metastatic disease is lung (83.4 %) followed by lymph node (49.7 %), intra-abdominal organ (29.7 %), kidney recurrence (25.5 %), bone (16.6 %) and other sites (11 %).

**Table 3:** Patient demographics and baseline clinical characteristics

	Number (%)		
	Pre-selection *N* = 30	Post-selection *N* = 115	Overall *N* = 145
Age (median)	19–68 (52)	28–77 (54)	19–77 (54)
Male	23 (76.7)	82 (71.3)	105 (72.4)
Nephrectomy	26 (86.7)	115 (100)	141 (97.2)
MSKCC risk			
Good	19 (63.3)	98 (85.2)	117 (80.7)
Intermediate	11 (36.7)	17 (14.8)	28 (19.3)
Poor	0	0	0
Heng risk			
Favourable	7 (23.3)	60 (52.2)	67 (46.2)
Intermediate	19 (63.3)	47 (40.9)	66 (45.5)
Poor	4 (13.3)	8 (6.9)	12 (8.3)
Pathology type			
Favourable	17 (56.7)	106 (92.2)	123 (84.8)
Other	13 (43.3)	9 (7.8)	22 (15.2)
No of met organ(s)			
1	10 (33.3)	42 (36.5)	52 (35.9)
2	7 (23.3)	41 (35.7)	48 (33.1)
3 +	13 (43.3)	32 (27.8)	45 (31.0)

Following HD IL2, 55 % of patients in this series were treated with VEGF-targeted therapies, 4 % enrolled into clinical trials, 2 % interferon and 22 % active surveillance. Seventeen percent had no available information.

### Response rate and survival analysis

**Fig. 2:**
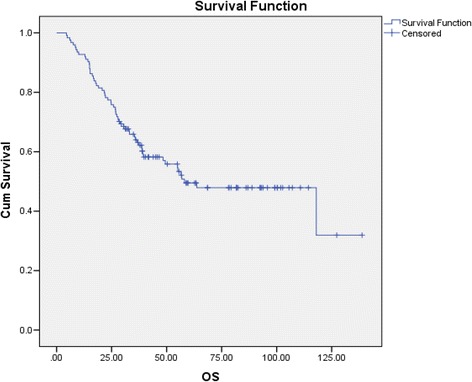
Kaplan –Meier curve showing overall survival (months) of patients with *Favourable* pathology

In total, there were 62 (42.8 %) responders in the entire cohort of which 30 (20.7 %) were complete. Seventy-six percent of CR remained relapse-free. ORR and CRR were 26.6 and 13.3 % prior to implementation of pathology selection (*N* = 30), these rose to 48.1 and 21.6 % respectively in the post-selection *Favourable* pathology group (Aug 2006-Jul 2013 *N* = 106). Median overall survival (OS) of the entire cohort was 49.4 months but was 58.1 months in the *Favourable* cohort (Fig. 2). Survival performance stratified by response is as shown in Additional file 1: Figure S1.

#### Histological subgroups and response

**Fig. 3:**
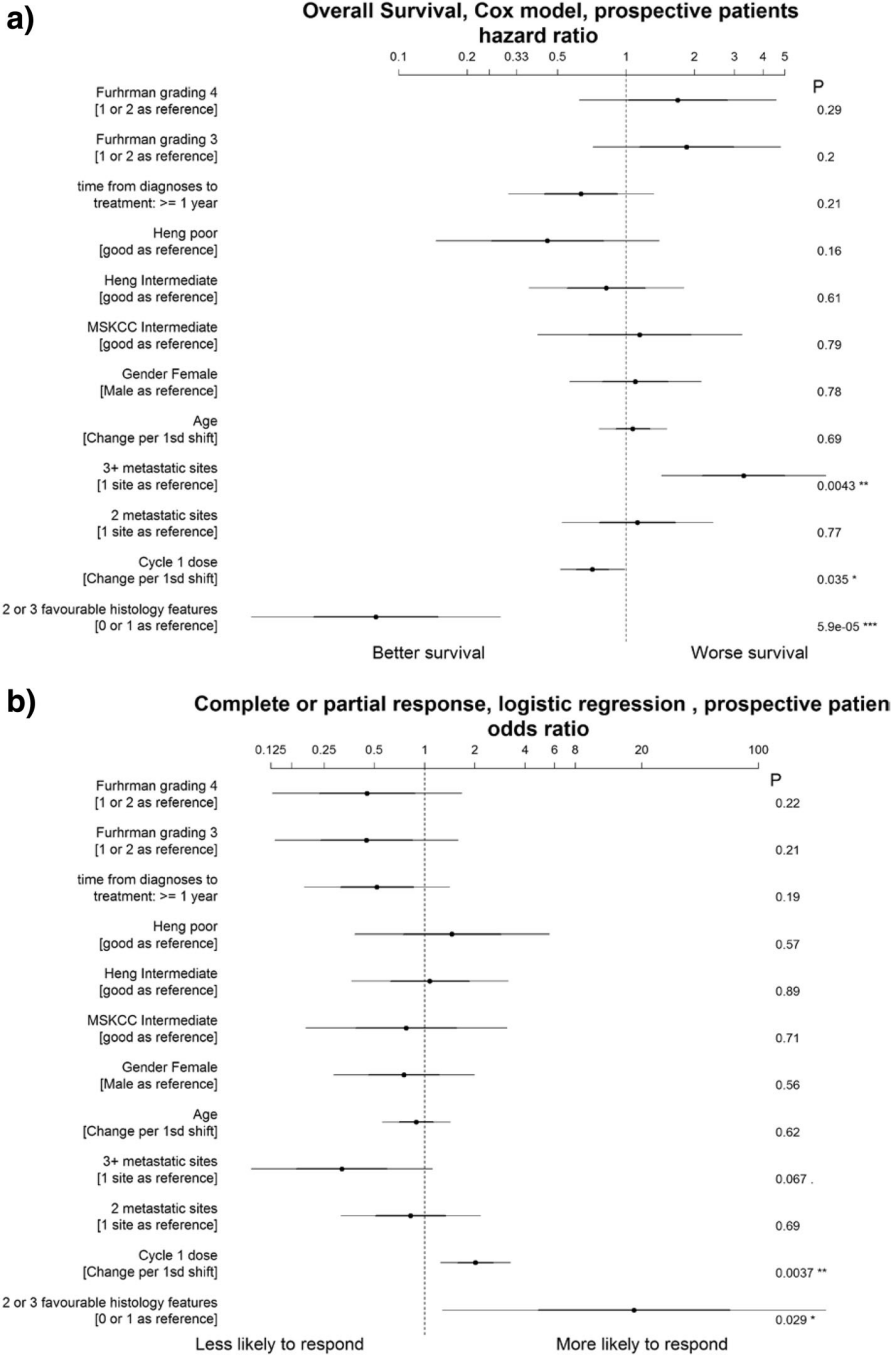
Forrest plots summarizing multivariate analysis and relationship between analyzed variables and (**a**) survival and (**b**) likelihood to response to IL2

We performed exploratory analysis on the various histology features as specified by our pathology selection criteria to study the response pattern further (Table 4). Response rate was highest in Group A where all favourable features were met (ORR 50.6 %; CRR 22.9 %). This cumulative benefit is confirmed using multivariate analysis where the presence of 2–3 favourable features is associated with about 20 times increase likelihood to respond to treatment than 0–1 (Fig. 3).

**Table 4:** Response analysis based on sub-classification of histological features among clear cell tumours

Number (%)	“Favourable” *N* = 123			“Other” *N* = 21			
	A *N* = 83	B *N* = 33	C *N* = 7	D *N* = 3	E *N* = 3	F *N* = 5	G *N* = 10
Papillary <10 %	✔	✔	✔	NO	NO	✔	NO
Alveolar or solid >50 %	✔	✔	NO	✔	NO	NO	NO
Granular <50 %	✔	NO	✔	✔	✔	NO	NO
ORR	42 (50.6)	13 (39.4)	3 (42.9)	2 (66.7)	0 (0)	0 (0)	1 (10.0)
CR	19 (22.9)	7 (21.2)	1 (14.3)	2 (66.7)	0 (0)	0 (0)	0 (0)
PR	23 (27.8)	6 (18.2)	2 (28.6)	0 (0)	0 (0)	0 (0)	1 (10.0)
SD	25 (30.1)	8 (24.2)	2 (28.6)	1 (33.3)	2 (66.7)	2 (40.0)	6 (60.0)
PD	16 (19.3)	12 (36.4)	2 (28.6)	0 (0)	1 (33.3)	3 (60.0)	3 (30.0)

Note: One patient with Type II Papillary carcinoma who achieved CR was excluded from this analysis

Notably, tumour with unfavourable alveolar but favourable solid features represented 31.5 % of all CRs in group A. Despite presence of unfavourable granular features in Group B which is the second largest subgroup, CRR was comparable to Group A (21.1 %). Independent of other histological criteria, patients whose tumour showed any favourable alveolar and/or solid features constituted highest proportion of CR (28/30, 93.3 %).

Response rate in *Other* category was much lower but there were 3 CRs. On review, 2 of the CRs both had >10 % papillary constituent (30 %) but favourable alveolar and granular features. Another CR was a non-clear cell tumour variant (Type 2 papillary carcinoma).

There were 6 patients whose tumour contained sarcomatoid architecture and responded to HD IL2 (of whom 2 were complete). All 6 had <10 % sarcomatoid constituents and also had 2 other favourable histological features. No response was seen at all with >10 % sarcomatoid element. Response amongst patients whose tumours contained <10 % papillary features were similar compared to none (Table 5).

**Table 5:** Response rate by proportion of papillary and sarcomatoid features

	None	<10 %	10–30 %	>30 %
Papillary	*N* = 105	*N* = 27	*N* = 3	*N* = 8
ORR	43 (40.9)	14 (51.9)	2 (66.7)	1 (12.5)
CRR	20 (19.0)	6 (22.2)	2 (66.7)	0
Sarcomatoid	*N* = 125	*N* = 12	*N* = 6	*N* = 2
ORR	56 (44.8)	6 (50.0)	0	0
CR	28 (22.4)	2 (16.7)	0	0

#### Membrane carbonic anhydrase 9 (CAIX) surface positivity

**Fig. 4:**
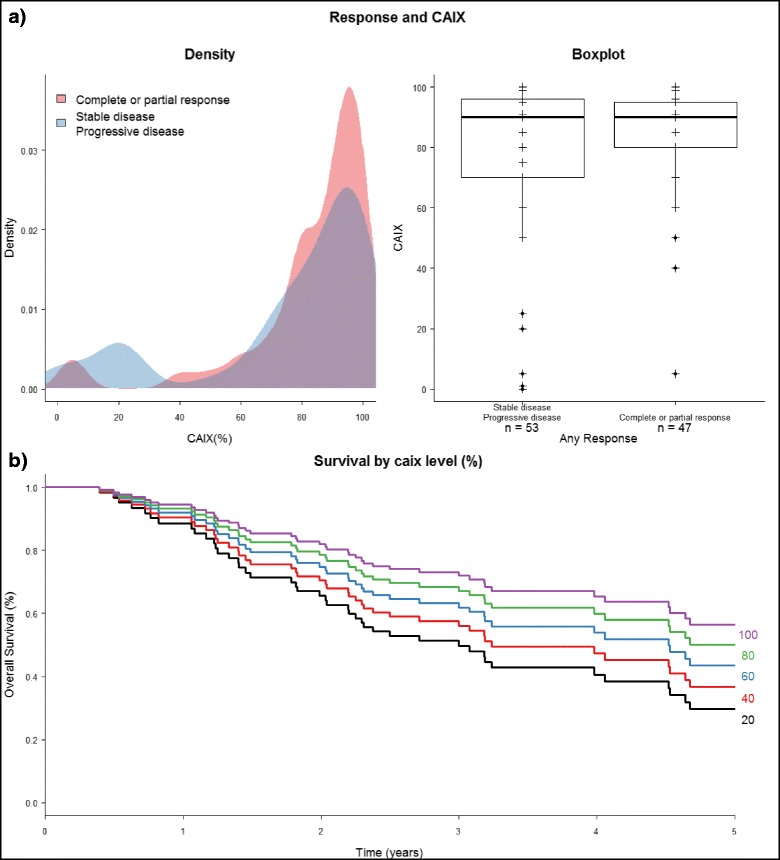
**a** Plots and curves showing no association between CAIX and any response **b** survival curve showing significant relationship with increasing CAIX expression

CAIX staining positivity was evaluable in 100 cases. CAIX surface positivity was compared between the responder group (CR and PR) and non-responder group (SD and PD). There was no difference in values between the groups. The median value was 90 in both groups and the distribution of values was similar (Fig. 4a) suggesting the lack of correlation to response. However, CAIX staining intensity appeared to have an incremental prognostic impact in this treatment cohort as shown on Fig. 4b.

#### Cycle 1 dose number

**Fig. 5:**
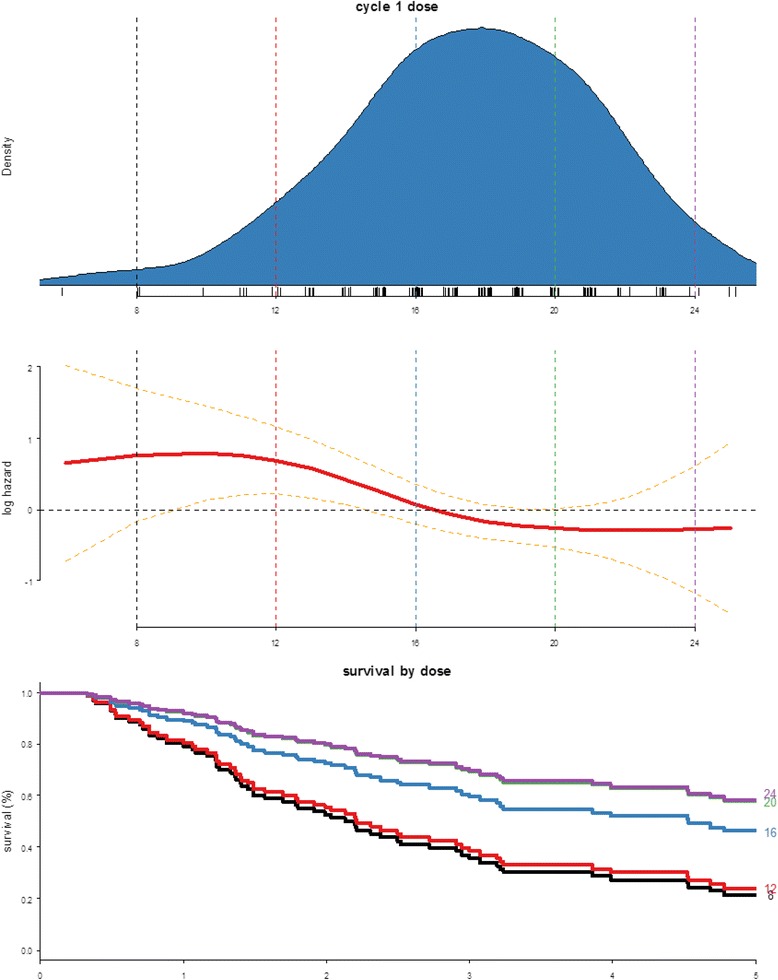
Relationship between cycle 1 dose intensity and survival probability

There is an incremental survival benefit with higher cycle 1 dose (between 12–20 total doses) (Fig. 5). Interestingly, the survival benefit seemed to tail off above 20 doses. Multivariate analysis also showed a significant association between higher cycle 1 dose intensity and any response (Fig. 3). There did not appear to be a significant association with complete response but this could be due to the smaller number of patients who attained CR.

#### Number of metastatic organ sites involvement

**Fig. 6:**
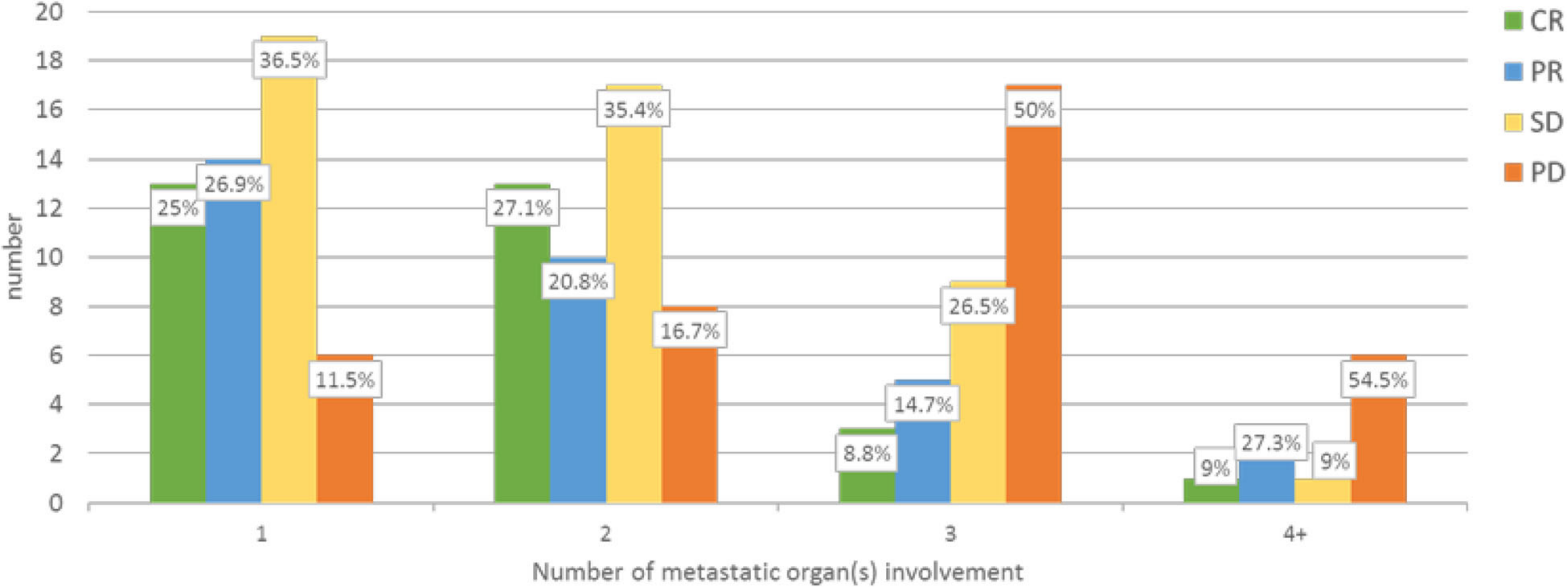
*Bar chart* showing distribution of responses according to number of metastatic organ sites

High proportions (86.7 %, 26/30) of CR cases were in patients with 1–2 number of organ sites (irrespective of number of metastatic lesions in each organ) involvement. The distribution of treatment response was similar between those with 1 and 2 organ site involvements. Therapeutic benefit of HD IL2 is significantly lower in those with 3 or more metastatic organ sites (Fig. 6) The inverse relationship between this variable and response is shown in multivariate analysis. This variable also has a clear impact on survival where patient with lesser number of involved organ(s) fared significantly better with median OS of 98, 43, 25 months for 1, 2, and 3 or more sites respectively (*p* = 0.001).

#### Other variables and prognostic factors

Time interval from diagnosis (of kidney cancer) to start of HD IL2 of less than 12 months, lower Fuhrman grading both showed a possible link to response but this was not statistically significant. There was no significant relationship between IL2 response rates with age or MSKCC risk group on univariate or multivariate analysis. Interestingly, evidence of immune mediated toxicity may be associated with response as, from our observation, 3 out of 4 patients with myocarditis, 12 (6 PR 6 CR) out of 18 patients with immune thyroiditis and 2/2 (both CR) with inflammatory arthritis responded to IL2.

#### Surgery for oligometastatic disease

Three complete responders had surgery after disease relapse, of which 2 remained disease-free and alive. Among patients with partial response, 2/15 who had surgery for residual oligometastatic disease remained disease – free by the end of our observation period.

### Treatment tolerance and toxicity

Treatment at our centre is administered on general oncology ward by experienced nurses, and local guidelines are in place to manage routine toxicity. Median number of cycles received in this series was 2 (Range 1–7). Median total number of IL2 doses received was 27 (Range 6–141). Median number of doses during cycle 1 was 18 (range 6–25). Majority of patients completed their treatment without significant complication. Most acute phase toxicities were short- lived and completely reversible.

All patients experienced anticipated grade 1–3 hypotension, tachycardia and fever secondary to vascular leak and cytokine release syndrome. Vascular leak syndrome was managed through the use of an intravenous fluid replacement sliding scale based on recorded blood pressure. Two patients developed a brief episode of grade 4 hypotension requiring less than 24 h inotropic support in intensive care unit.

Cardiac complications were seen in 15 patients. Four patients developed treatment-related myocarditis as proven by troponin rise and cardiac imaging. Treatment was discontinued in all cases. All received cardiology input with 2 patients receiving short-term supportive treatment with a combination of beta-blockers and angiotensin-converting enzyme inhibitors. Cardiac function returned to pre-treatment levels in 3/4 affected patients. One patient has ongoing mild left ventricular impairment. One patient, in addition to myocarditis developed a secondary cardiac thrombus and embolic stroke. This was managed conservatively with anticoagulation and subsequently completely recovered with no residual neurological deficit. One patient suffered an acute coronary event with associated ECG changes and troponin rise. This was successfully treated with coronary angioplasty with no significant cardiac dysfunction. The patient ultimately obtained a complete remission after completing treatment with reduced dose IL2.

Ten patients developed supraventricular cardiac tachy-arrhythmias with atrial fibrillation being the commonest rhythm. Five patients required chemical cardioversion with intravenous amiodarone whilst two others were rate-controlled with a combination of digoxin and bisoprolol. All patients reverted to normal sinus rhythm within 48 h.

Eighteen patients developed immune-related thyroiditis. One patient required short term beta-blockade for symptomatic hyperthyroidism. All affected patients subsequently developed hypothyroidism requiring long-term thyroid replacement.

Two patients developed grade 2 arthritis (seronegative), both required short-term treatment with anti-inflammatory and immunomodulatory treatment (cyclooxygenase-2 inhibitor and hyrdroxychloroquine). All inflammatory symptoms fully resolved in both cases and both were able to complete treatment achieving durable complete responses. One patient was, however, left with a finger deformity which may require subsequent surgery. No patients died as a direct consequence of HD IL2 treatment.

## Discussion

The use of immunotherapy such as checkpoints inhibitors is increasingly promising in the treatment of mRCC in recent times. As we continue to explore the exciting application of newer agent such as Nivolumab, findings from our review shows that HD IL2 remains an important treatment option for this disease group. Despite being an approved treatment option for mRCC for many years, uptake of HD IL2 has been low due to the complexity of therapy (requiring expert specialist management), its significant toxicity and modest response rate in unselected population. The rise of VEGF (Vascular Endothelial Growth Factor)-targeted therapy with more predictable efficacy and manageable side effects further reduced the appeal of HD IL2 as first-line treatment in recent times. Unfortunately, the inevitable tumour resistance associated with targeted therapies meant that therapeutic intent remains palliative for the majority.

The longstanding difficulty with HD IL2 has been the lack of ability to pinpoint the subset of patients who can benefit significantly from this treatment. There were previous reports suggesting unfavourable IL2 outcome in patients with higher disease burden (more than one metastatic site), and short progression free interval of less than 1 year [16]. Other observations possibly associated with tumour response includes development of thrombocytopenia [17], thyroid dysfunction [18], rebound lymphocytosis [19], and low monocytes and granulocytes [20]. However, these are post-treatment findings and therefore not helpful in pre-treatment patient selection.

The plausible link between pathology and response shown by Upton and colleague as well as our own results has increased optimism for this challenging treatment option. The maintained high efficacy level in our post-pathology selection cohort demonstrated by this updated report continues to uphold this hypothesis. In our case series, patients with tumours that met all three pathology criteria fared extremely well, with 1 in 2 responding to treatment and 1 in 4–5 achieving CR. This significant cumulative predictive benefit based on higher number of favourable pathology criteria met was shown in our multivariate analysis. We postulate that a high level of alveolar features may be more important than other features as previously discussed. We also postulate that the presence of high solid pattern is a favourable feature. In our experience, the presence of papillary or sarcomatoid architecture of less than 10 % is not necessarily unfavourable and should not be excluded if there were also other good histological features. Based on our experience here, an upper limit of 30 % for papillary pattern may be considered an appropriate cut-off for future cases provided other features are favourable.

We note the result from the prospective biomarker validation study by the Cytokine Working Group (SELECT) which did not show any significant predictive value of their proposed pathology selection tool also termed Integrated Selection Model (ISM) [21]. Although the response rate in SELECT was considered better (ORR 25 %) than historical result, this was nevertheless lower than that predicted by the author (30–40 %) for the required statistical power of the trial. Interestingly, the published information suggested only a very low proportion (<10 %) with favourable (>50 %) alveolar architecture within the SELECT population, and based on our findings showing correlation of this feature to response, this could be one of the likely factor for the lower than expected response rate in SELECT thus could explain the lack of detectable effect. Importantly, the ISM did not consider solid features to be a favourable feature whereas our data convincingly show it to be one. In addition, CAIX staining positivity was integrated in the categorisation of ISM which, given its lack of predictive value shown in our report, may also have an impact on the overall outcome. Thus we feel the SELECT trial may not have used optimal selection and the result from this trial should not discourage further exploration and utilisation of tumour pathology as predictive biomarker in HD IL2.

The development of cancer immunotherapy has been quite remarkable in the last 5 years where impressive clinical benefits are seen across various tumour sites. With rising healthcare cost and emphasis on personalised care, there is an urgent need for a clinically relevant biomarker to better select patients for a particular type of treatment. Example biomarkers of interest currently includes cell surface molecules such as PDL1, CD70 and ICOS expression, tumour mutational load by exome sequencing, as well as detailed immune cells analysis by immune profiling or by TCR sequencing. As yet, none of these has been validated for clinical use and some findings have been highly inconsistent - this applies particularly to PDL1 expression where the low level of expression and the dynamic nature of its expression seem likely to limit its value. However, the greater understanding of cancer immunotherapy and the wider availability of molecular immune assessment (including mutational analysis of the tumour, TCR sequencing and potentially identification of immunogenic targets) could potentially facilitate refinement of our selection criteria to further improve the therapeutic advantage. At present, based on our maintained high response rates in the post-selection era, tumour morphology represents the most sensitive and reliable predictor of IL2 response to date and may well have similar predictive effect in other newer immunotherapies.

Additionally, there was evidence of response and survival benefit with higher number of IL2 doses received during cycle 1 in this cohort. Similar effect was also previously observed by another group using HD IL2 in malignant melanoma [17]. This is a relatively independent and thus useful variable compared to total number of overall doses or treatment cycles which is inherently dictated by response, i.e. patient who responded will naturally do better and also continue to receive more treatment. It is however possible that although all patients offered this treatment were considered fit, there may be other likely unaccounted variables that could have affected treatment tolerance or even survival. There also seems to be a loss of effect above 20 doses, although this could be a true effect, there is a high degree of uncertainty above this threshold due to the small number of patients receiving more than 20 doses. Even with such possible limitation, this remains a convincing finding, and striving for maximal tolerance within safe limits especially during first cycle appears to be a justifiable strategy to improve outcome.

The number of metastatic organ sites also had a significant bearing on response and survival in this patient series. This is in keeping with previous reports, and may be associated with increased heterogeneity allowing tumour escape or immune suppressive effects of heavy tumour burden [12, 16]. Limiting treatment only to those with 1–2 metastatic organ sites could avoid unnecessary toxicity in those who are less likely to respond.

The treatment tolerance and toxicity was within expectation. Importantly, all adverse effects were reversible (except thyroid dysfunction) and there has been no treatment related death in this cohort. In keeping with previous reports, development of immune related events may be associated with better outcome as shown by observation of good proportion of responders among those affected by these phenomena (thyroiditis, myocarditis and arthritis).

## Conclusion

Our analysis has unveiled interesting clinical and treatment-related factors linked to better response and survival, which could potentially be exploited to enhance outcomes further when using HD IL2. A good proportion of well-selected patients can benefit from durable treatment-free remission when complete response is achieved, a unique advantage compared to all other currently available options which require continuous treatment. Fit mRCC patients especially those with favourable pathology and low disease burden should be referred for consideration of HD IL2 at an experienced centre. Patient selection based on tumour morphology has helped achieve much-improved HD IL2 efficacy, and remains the cornerstone of our practice. Effort to gain a better understanding of this interesting relationship through continued translational research will refine and enhance the use of immunotherapy in mRCC further. The use of HD IL2 in combination with other immunomodulatory therapies such as checkpoint inhibitors should also be explored to investigate if higher rates of complete remission can be obtained. We hope our experience and information shared here will encourage oncologists to recommend this therapy with renewed confidence.

## Additional file

Additional file 1: Figure S1. (a) Survival curve showing overall survival by response to HD IL2 and (b) Percentage of survival by response at 1, 3 and 5-years. (DOCX 80 kb)

## Abbreviations

CR: Complete response
CRR: Complete response rate
HD IL2: High dose interleukin 2
ICOS: Inducible costimulatory
mRCC: Metastatic renal cell carcinoma
MSKCC: Memorial Sloan-Kettering Cancer Centre
NHS: National Health Service
ORR: Overall response rate
PD: Progressive disease
PD1: Programmed cell death 1
PDL1: Programmed death-ligand 1
PR: Partial response
SD: Stable disease
TCR: T cell receptor
ULN: Upper limit of normal
VEGF: Vascular endothelial growth factor
