# Supplementary figures and images for: High-dose interleukin2 – a 10-year single-site experience in the treatment of metastatic renal cell carcinoma: careful selection of patients gives an excellent outcome
Source: J Immunother Cancer. 2016 Oct 18;4:67. doi: 10.1186/s40425-016-0174-5 (PMC5067981; doi:10.1186/s40425-016-0174-5)

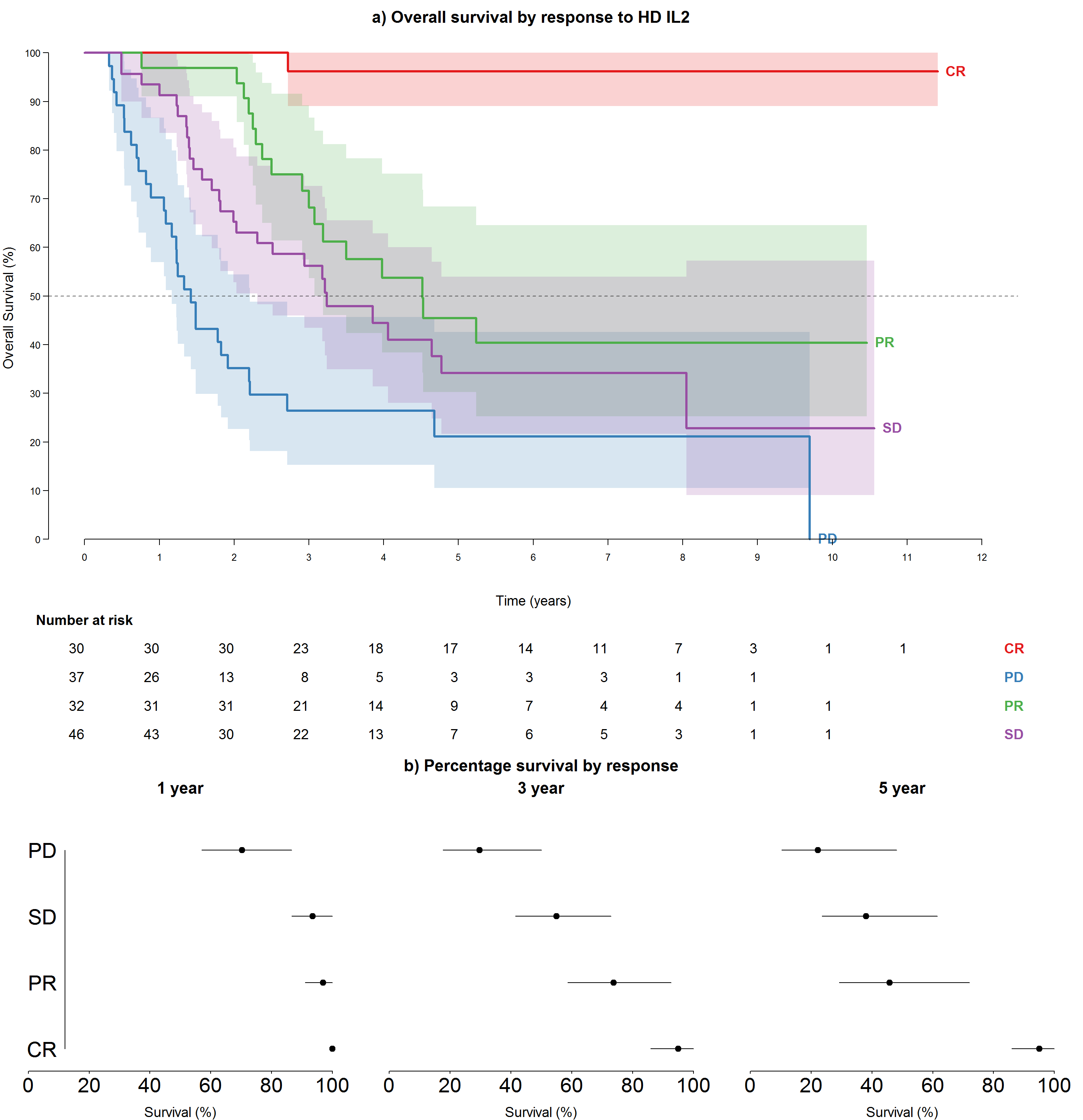

Supplement: Additional file 1: Figure S1. — (a) Survival curve showing overall survival by response to HD IL2 and (b) Percentage of survival by response at 1, 3 and 5-years. (DOCX 80 kb) [file 40425_2016_174_MOESM1_ESM.docx]
